# Immune-inflammatory and hypothalamic-pituitary-adrenal axis biomarkers are altered in patients with non-specific low back pain: A systematic review

**DOI:** 10.3389/fimmu.2022.945513

**Published:** 2022-09-02

**Authors:** Juan P. Sanabria-Mazo, Ariadna Colomer-Carbonell, Meritxell Carmona-Cervelló, Albert Feliu-Soler, Xavier Borràs, Mar Grasa, Montserrat Esteve, Michael Maes, Sílvia Edo, Antoni Sanz, Juan V. Luciano

**Affiliations:** ^1^ Institut de Recerca Sant Joan de Déu, Esplugues de Llobregat, Spain; ^2^ Teaching, Research and Innovation Unit, Parc Sanitari Sant Joan de Déu, St. Boi de Llobregat, Spain; ^3^ Faculty of Psychology, Autonomous University of Barcelona, Cerdanyola del Vallès, Spain; ^4^ Faculty of Biology, University of Barcelona, Barcelona, Spain; ^5^ Biomedical Research Centre in Physiopathology of Obesity and Nutrition (CIBERobn), Institute of Health Carlos III, Madrid, Spain; ^6^ Department of Psychiatry, Faculty of Medicine, Chulalongkorn University, Bangkok, Thailand

**Keywords:** non-specific low back pain, immune-inflammatory biomarkers, cytokines, hypothalamic-pituitary-adrenal axis, cortisol

## Abstract

This systematic review aimed to investigate immune-inflammatory and hypothalamic-pituitary-adrenal (HPA) axis biomarkers in individuals with non-specific low back pain (NSLBP) compared to healthy control. The search was performed in five databases until 4 November 2021. Two reviewers independently conducted screenings, data extraction, risk of bias, and methodological quality assessment of 14 unique studies. All studies reported the source of the fluid analyzed: nine studies used serum, two used plasma, one used serum and plasma, and two studies used salivary cortisol. We found preliminary and limited evidence (only one study for each biomarker) of increased levels in growth differentiation factor 15 (GDF-15), interleukin-23 (IL-23), transforming growth factor–beta (TGF-β), and soluble tumor necrosis factor receptor 1 (sTNF-R1) in NSLBP. Inconsistent and limited evidence was identified for interleukin-10 (IL-10). Although C-reactive protein (CRP), interleukin-6 (IL-6), and tumor necrosis factor–alpha (TNF-α) levels appear to increase in NSLBP, only one study per each biomarker reported statistically significant differences. Interleukin-1 beta (IL-1β), interleukin-17 (IL-17), interferon gamma (IFN-γ), and high-sensitivity CRP (hsCRP) showed no significant differences. Regarding cortisol, one study showed a significant increase and another a significant decrease. More robust evidence between GDF-15, IL-23, TGF-β, and sTNF-R1 with NSLBP is needed. Moreover, contrary to the findings reported in previous studies, when comparing results exclusively with healthy control, insufficient robust evidence for IL-6, TNF-α, and CRP was found in NSLBP. In addition, cortisol response (HPA-related biomarker) showed a dysregulated functioning in NSLBP, with incongruent evidence regarding its directionality. Therefore, our effort is to find adjusted evidence to conclude which immune-inflammatory and HPA axis biomarkers are altered in NSLBP and how much their levels are affected.

**Systematic Review Registration:**

https://www.crd.york.ac.uk/prospero/display_record.php?ID=CRD42020176153, identifier CRD42020176153.

## Introduction

Non-specific low back pain (NSLBP) is an umbrella term that refers to the presence of tension, soreness, or stiffness in the lower back region that cannot be attributed to any recognizable pathology or be considered as a symptom of another disease (1, 2). Depending on the pain duration episode, NSLBP can be classified as acute (less than 6 weeks), subacute (between 6 and 12 weeks), and chronic (12 weeks or more) pain (3). Currently, NSLBP is recognized by the World Health Organization as the greatest contributor to global disability worldwide (4). The lifetime prevalence is between 70% and 90%, and its point prevalence is about 10% (5). The risk of suffering this health condition increases in elder populations, women, smokers, and people with obesity (6). The constant pain experience affects the physical and psychological patients’ wellbeing (7), being considered a public health problem with a great economic burden at the societal level (8).

Although the etiopathogenesis of NSLBP involves several mechanisms, there are multiple physical and psychosocial factors that play a role in the onset and recurrence of NSLBP (9). Research has also highlighted that central sensitization—an enhancement in the neural signaling within central nociceptive pathways due to neuroplastic mechanisms—may play a role in the development and maintenance of this pathology (10). In recent years, there has been burgeoning interest in the interplay of the immune system and hypothalamic-pituitary-adrenal (HPA) axis function under chronic and non-chronic pain conditions (11, 12). Cytokines related to the immune-inflammatory response system (IRS) (13), such as interleukin-1 beta (IL-1β) or interleukin-6 (IL-6), have been involved in central sensitization (14, 15). Systemic inflammation has been shown to be a key factor in NSLBP, as well as elevated levels of IRS biomarkers increase neuropathic and inflammatory pain (15). Furthermore, stressful events (16), poor sleep (17), obesity (18), anxiety (19), and depression (20)—elements which are usually over-represented in patients experiencing chronic pain—are accompanied by alterations of the IRS.

Pain is associated with a dysregulation of the stress-response system (16, 21). Being exposed to a painful condition is a potential stressor that may result in a triggered cortisol response (11, 22). Under normal conditions, cortisol binds to the glucocorticoid receptor (GR), present on immune cells, and acts as anti-inflammatory, but sustained increased levels of cortisol block cortisol binding with GR as a compensatory down-regulation system and may disrupt the negative feedback mechanism by which cortisol normally inhibits the continued release of corticotropin-releasing hormone by the hypothalamus, events leading to a pro-inflammatory response (23). Moreover, HPA axis dysfunctions including alterations in cortisol (24–26) and adrenocorticotropic hormone (20) are associated with greater pain levels under chronic and neuropathic conditions (15, 27).

While some studies have explored the presence of altered immune-inflammatory biomarker levels in NSLBP, it remains uncertain how the functioning of the IRS and HPA in NSLBP differs from the healthy population. Nonetheless, given the methodological approach of previous systematic reviews, nowadays, some immune-inflammatory biomarkers that could potentially be associated with NSLBP severity can be targeted. Firstly, the systematic review by van den Berg et al. (28) provided evidence for a positive association between C-reactive protein (CRP) and IL-6 levels and severity of NSLBP, as well as an association between tumor necrosis factor**–**alpha (TNF-α) and the presence of NSLBP. Secondly, the systematic review by Morris et al. (29) reported elevated CRP in non-specific acute low back pain (acute NSLBP) and increased TNF-α in non-specific chronic low back pain (chronic NSLBP). Finally, the systematic review by Lim et al. (30) informed of elevated CRP, TNF-α, and IL-6 levels in NSLBP.

The aforementioned systematic reviews used a less restrictive selection of articles, including those with heterogeneous comparison groups [not exclusively healthy controls (HC)] or even without a comparison group. The current research tries to disentangle which immune-inflammatory biomarkers are altered in NSLBP by reviewing articles using a pure HC comparison group. This effort underlies the necessity to clarify possible misleading findings previously reported in the NSLBP field such as CRP, TNF-α, and IL-6 associated with NSLBP severity (28–30) as well as CRP and TNF-α with acute NSLBP and chronic NSLBP, respectively (29). Crucially, the use of a specific tool for assessing the methodological quality (MQ) of aimed studies at exploring immune-inflammatory biomarker differences was incorporated for the first time to guarantee adjusted and precise results. Finally, unlike any previous systematic review that focused on NSLBP, the current research also evaluated preliminary evidence of alterations in peripheral levels of HPA-related biomarkers. The interplay role between HPA-related biomarkers and immune-inflammatory biomarkers was not explored in previous systematic reviews.

This systematic review aimed to investigate immune-inflammatory and HPA biomarkers in individuals with NSLBP compared to HC. The identification of signature biomarkers might help to deepen our understanding of the biological pathways underpinning NSLBP.

## Materials and methods

### Protocol and registration

This systematic review was performed according to the Preferred Reporting Items for Systematic Reviews and Meta-Analyses statement (PRISMA) (31). The review protocol was registered at PROSPERO (registration number: CRD42020176153).

### Data sources and searches

Systematic searches were conducted in PubMed, Medline^®^, Web of Science, PsycINFO, and Scopus. The search strategy identified studies that included combinations of the main terms [(back pain) OR (lumbar vertebrae) OR (sciatica) OR (radiculopathy)] AND the specific terms of biomarkers [(inflamm*) OR (cytokines) OR (tumor necrosis factor) OR (transforming growth factor–beta) OR (C-reactive protein) OR (cortisol) OR (hypothalamic pituitary adrenal)] found as keywords in all fields, titles, or Medical Subject Headings (MesH). The search terms were selected according to a validation by a panel of experts and a review of the search strategies used in previous systematic reviews on immune-inflammatory biomarkers (28–30, 32) and HPA axis (22, 33, 34).

The specific Boolean searches were adjusted according to the Peer Review of Electronic Search Strategies (PRESS) guideline statement (35). The format of each database is presented in **Appendix A**. The following limits and filters were activated in all databases if possible: publication date (from 1 January 2005 to 4 November 2021), type of publication (only studies of interest), species (humans), and languages (English and Spanish). The publication date was defined considering the results obtained in previous systematic reviews. Moreover, it was continuously updated through the immediate alerts of the selected databases. The reference list of included papers was examined by a reverse citation search for further analysis. In addition, reviews, as well as gray literature (a search carried out in Google Scholar), were used to verify the reference list in order to ensure that all eligible studies were included.

### Eligibility criteria

The selection of the eligibility criteria was performed considering the recommendations of the PICOS strategy (36). The inclusion criteria were (a) adults (≥18 years of age) with primary NSLBP; (b) screening of acute (up to 6 weeks), subacute (from 6 to 12 weeks), or chronic (more than 12 weeks) NSLBP according to medical history (3); and (c) baseline data from experimental, quasi-experimental, case-control, and observational studies, with a HC (defined by the absence of any significant disease, including chronic pain).

The exclusion criteria were (a) secondary pain or low back pain associated with a range of serious conditions (e.g., fracture, systemic or inflammatory diseases, cauda equina syndrome, active infection, scoliosis, post-surgical, modic changes, radiculopathy, or cancer); (b) the presence of a severe psychiatric disorder (e.g., bipolar disorder or schizophrenia), substance dependence/abuse, and neuro-inflammatory/neurodegenerative disorders (e.g., stroke, multiple sclerosis, or Alzheimer’s dementia), and immune-inflammatory disorders (e.g., rheumatoid arthritis, inflammatory bowel disease, or atherosclerosis); (c) no measurement of immune-inflammatory biomarkers or cortisol as an outcome; (d) non-original (editorials, guidelines, systematic reviews, protocols, etc.), clinical trials, case reports, and qualitative studies; (e) animal or post-mortem studies; and (f) no full-text available.

### Data management and study selection

In the first phase, search results from all databases were exported to Mendeley. In the second phase, duplicate articles in the databases were removed automatically by Mendeley and manually by the first reviewer (JS-M). In the third phase, the file was blind-screened in Rayyan (37) based on their titles and abstracts by the first reviewer (JS-M) and the second reviewer (MC-C). In the fourth phase, the reviewers exported the references selected in Rayyan QCRI to a form developed by the research team and blind-screened the full texts to check study eligibility. In the fifth phase, extraction of data from selected full text, risk of bias (RoB), and MQ of included studies was carried out by the reviewers, being key information from each study entered into a standardized data extraction form.

### Data extraction and study coding

The first reviewer (JS-M) summarized all the relevant information from the studies in a spreadsheet, whereas the second reviewer (MC-C) checked the extracted data in order to avoid errors in information recording. The general information extracted from each study was authors, objective, type of research, year of publication, location, diagnostic criteria, duration of illness, clinical data, sample size in the study groups (NSLBP and HC), age, body mass index (BMI), gender (% of women), and type of assay used to quantify biomarker levels. The biomarker information extracted from each study was sample size (n), mean (M), standard deviation (SD), effect size (using Hedges g), and statistical significance between groups (*p*-value).

### Risk of bias and methodological quality

RoB was assessed using an adapted version of the National Heart, Lung, and Blood Institute tool (38). The total score was divided into low (8–10), medium (4–7), and high (≤3) RoB based on a scale from −10 to 10. The answer options were scored with a 1 when the criterion was met, with a 0 when it was unclear or cannot be determined, and with −1 when it was not reported.

MQ was assessed according to the Immune Confounders Scale (ICS; **Appendix B**) (32) adapted to also take into account potential biomarkers of the HPA axis. This checklist comprises two parts: (a) 10 questions for the assessment of the quality biomarkers’ evidence, considering the most important analytical aspects (e.g., sample size, matched groups, and reporting detection limit), and (b) 20 questions for the consideration of possible critical confounders that could induce heterogeneity in the results of the studies analyzed. The first part, in which questions were scored with a 1 if evidence of compliance was reported, is calculated on a scale from 0 to 10 with higher scores indicating better MQ (0–4: low; 5–7: medium; and 8–10: high quality). The second part of the ICS is on a scale from 0 to 29, where 0 indicates the total control of all confounding variables (e.g., matching study groups, meeting all exclusion criteria, or statistically adjusting for background variables) and 29 indicates the total absence of control.

### Data analyses

All first and corresponding authors of selected articles with missing data for the synthesis of this systematic review were contacted by email in two rounds.

## Results

### Selection and inclusion of studies

**Figure 1:**
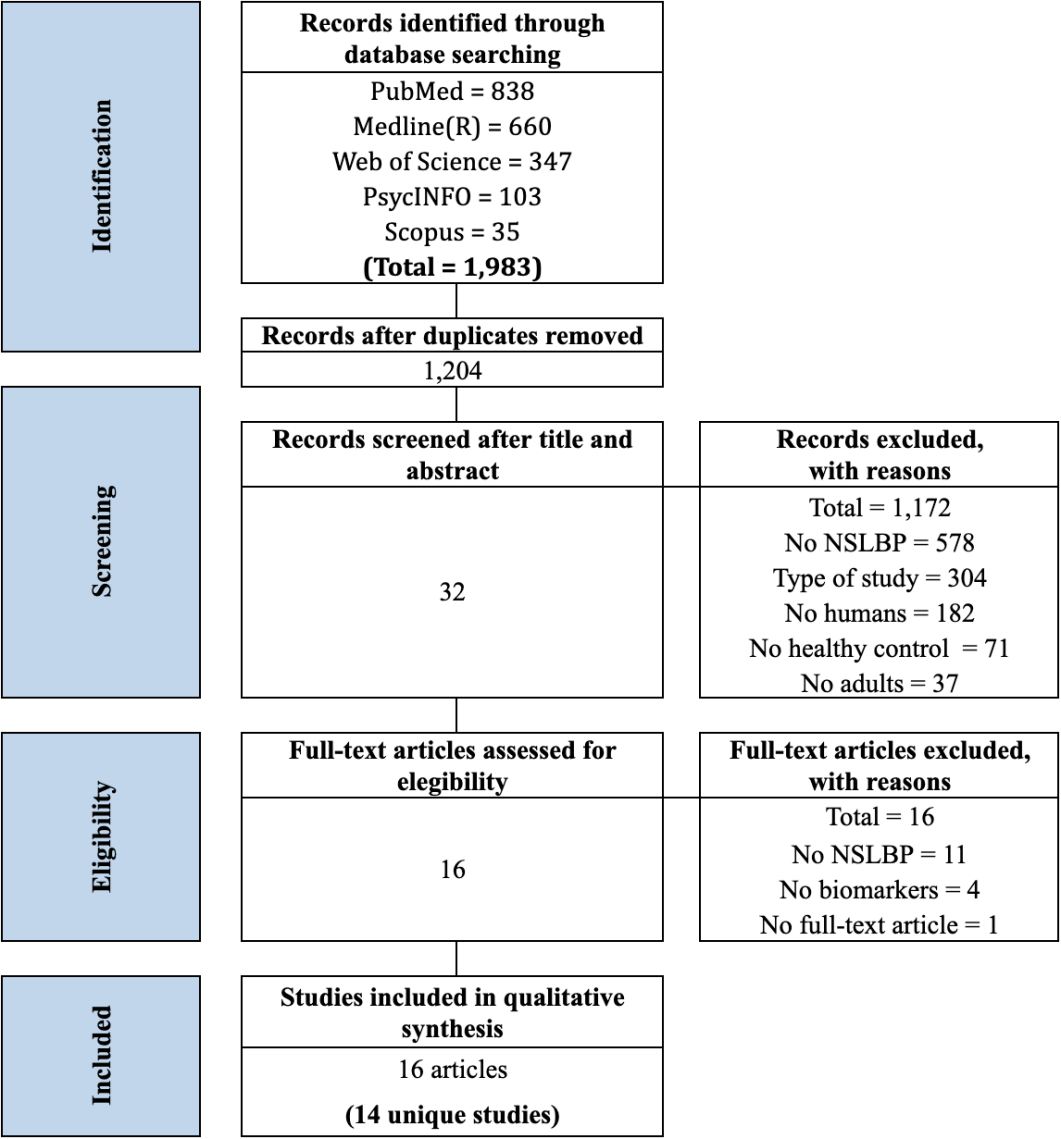
Preferred Reporting Items for Systematic Reviews and Meta-analyses (PRISMA) flowchart from record identification to study inclusion.

**Figure 1** displays the flowchart of the study selection process. The initial search conducted in the five databases yielded up to 1,983 potentially relevant studies. In the first phase, after the removal of duplicates, 1,204 titles and abstracts were screened, of which 32 studies were selected for full-text revision with a 99% preliminary consensus. We excluded studies not focused on NSLBP (n = 578), not the type of targeted study (n = 304), not human studies (n = 182), not including a HC group (n = 71), and not adults (n = 37).

In the second phase, after the full-text screening, 16 studies were excluded with an 87% of preliminary consensus. We excluded studies with participants with a different diagnosis from NSLBP (n = 11), not including any immune-inflammatory biomarkers or cortisol levels (n = 4), and without a full-text available (n = 1). Finally, 16 articles were included in this systematic review, reporting on 14 unique studies. When studies derived from the same sample were identified [e.g., Wang et al. (19) and Wang et al. (20) and Klyne et al. (39), Klyne et al. (40), and Klyne et al. (41)], the one with the largest sample was selected [(Wang et al. (19) and Klyne et al. (40)]. There was no need for a third reviewer to resolve a disagreement in any of the screenings.

### Characteristics of the included studies

**Table 1** shows the characteristics of the 14 included studies. The sample size of the studies groups ranged from 11 to 556 in NSLBP and 7 to 1,572 in HC, and the mean age ranged from 21 to 75 years old and 20 to 75 years old in NSLBP and HC, respectively. A total of 10 studies assessed individuals with chronic NSLBP, one with acute NSLBP, and three with NSLBP (without stipulating the duration). Illness duration was reported in five studies, ranging from 20 to 88 months, and BMI was reported in nine studies, ranging from 19 to 48 kg/m^2^. The included studies were conducted in eight countries: Germany (n = 6), USA (n = 2), Canada (n = 1), Australia (n = 1), Brazil (n = 1), Norway (n = 1), Israel (n = 1), and China (n = 1). Study designs included six quasi-experimental, five case-control, two observational, and one experimental.

**Table 1:** Characteristics of the included studies (*n* = 14).

Author (year)	Country	Design	Diagnosis	*n*		Age (SD or Range)		Female, %		BMI (SD or Range)		Pain duration, months	Assessed biomarkers
				NSLBP	HC	NSLBP	HC	NSLBP	HC	NSLBP	HC		
*Immune-inflammatory biomarkers*													
Gebhardt et al. (2006) (42)	Germany	CC	NSCLBP	41	1572	42.2 (27-57)	NR (20-64)	65.8	NR	27.7 (19-48)	NR	24.3	hsCRP
Wang et al. (2008) (43)	Germany	CC	NSCLBP	120	120	46.6 (10.9)	45.4 (11.4)	43.3	43.3	26.1 (18-35)	27.1 (19-48)	NR	TNF-α
Wang et al. (2010) (20)	Germany	CC	NSCLBP	58	29	44.7 (24-68)	40.8 (23-66)	58.6	58.6	24.2 (18-33)	27.1 (19-48)	20.4	TNF-α
Roy et al. (2010) (27)	Canada	QE	NSCLBP	11	10	45.6 (8.9)	47.5 (16.2)	36.36	40.0	28 (3.7)	25.3 (3.6)	NR	hsCRP, IL-6
Heffner et al. (2011) (44)	USA	CC	NSCLBP	25	25	30.8 (11.4)	30.8 (11.4)	60.0	60.0	NR	NR	NR	IL-6
Luchting et al. (2014) (45)	Germany	QE	NSCLBP	37	25	44.5 (21-73)	43.0 (24-54)	62.2	52.0	NR	NR	70.1	IL-6, IL-10, IL-17, IL-23
Queiroz et al. (2015) (46)	Brazil	QE	NSLBP	71	71	71.4 (5.06)	71.5 (4.87)	100	100	30 (4.8)	27.5 (4.4)	NR	sTNF-R1, IL-6
Luchting et al. (2016) (47)	Germany	QE	NSCLBP	19	19	47.0 (13)	40.0 (11)	79.0	58.0	23.9 (3.1)	23.6 (2.9)	71.8	IL-1β
Li et al. (2016) (48)	China	QE	NSCLBP	35	35	NR (45-75)	NR (45-75)	NR	NR	NR	NR	NR	IL-6, IL-10
Degenhardt et al. (2017) (49)	USA	EX	NSLBP	33	7	37.7 (11.7)	32.0 (9)	72.7	71.0	25.4 (4.3)	24.1 (4.3)	NR	IL-1β, IL-6, TNF-α, CRP
Klyne et al. (2018) (40)	Australia	CC	NSALBP	109	55	29.0 (8)	27.0 (6)	46.8	69.1	24.1 (3.7)	22.9 (4.1)	NR	CRP, IL-6, IL-1β, TNF-α
Tarebeith et al. (2019) (50)	Israel	OB	NSLBP	556	522	46.3 (NR)	39.6 (NR)	54.64	54.64	29 (NR)	27 (NR)	NR	GDF-15
*HPA axis markers*													
Muhtz et al. (2013) (24)	Germany	QE	NSCLBP	20	33	44.9 (14.6)	33.3 (12.0)	60	63.6	NR	NR	87.7	Cortisol	
Sveinsdottir et al. (2015) (26)	Norway	OB	NSCLBP	305	845	44.0 (9.34)	46.0 (9.7)	54	50	NR	NR	NR	Cortisol	

BMI, body mass index (kg/m^2^); CC, case control; EX, experimental; OB, observational; QE, quasi-experimental; HC, healthy controls; NSLBP, non-specific low back pain; NSSLBP, non-specific subacute low back pain; NSCLBP, non-specific chronic low back pain; NSALBP, non-specific acute low back pain; NR, not reported; CRP, C-reactive protein; GDF, growth differentiation factor; hsCRP, high-sensitivity CRP; IL, interleukin; TNF, tumor necrosis factor.

We found that 100% of the included studies indicated the type of sample (plasma, serum, or saliva), 57% were free of pain medication, 36% were free of mood medication including antidepressants and mood stabilizers, 50% had matched participants by age and gender, and 43% specified the time of extraction. Approximately 93% reported the diagnostic criteria for NSLBP, 36% assessed the pain intensity level, 29% calculated the severity of depressive symptomatology, and 29% measured the back functional status. Regarding outcomes, approximately 43% assessed more than one immune-inflammatory biomarker and 14% assessed cortisol. Eighty-six percent of the studies reported the name of the manufacturer of the test kits used to assay the biomarkers. The characteristics of the individual studies are detailed in **Table 1**.

### Risk of bias assessment and methodological quality

Applying the National Heart, Lung, and Blood Institute tool (38), we found that 29% of the studies presented low and 71% moderate RoB. Regarding the quality of the studies, 64% presented medium and 36% presented low MQ. Additionally, 42% of the studies did not control for at least 10 of the 20 established confounder variables. There was a 99% agreement in the RoB assessment and 91% in the MQ assessment. A third reviewer was not needed for reaching a consensus. The RoB assessment is presented in **Table 2**, whereas the MQ assessment is reported in **Appendix C**.

**Table 2:** Risk of bias assessment using an adapted version of the National Heart, Lung, and Blood Institute tool for included studies (*n* = 14).

Study	NIHLBI										Score	RoB
	Q1	Q2	Q3	Q4	Q5	Q6	Q7	Q8	Q9	Q10		
**Immune-inflammatory biomarkers**												
Gebhardt et al. (2006) (42)	1	1	1	1	−1	1	1	1	1	−1	6	Moderate
Wang et al. (2008) (43)	1	1	−1	1	−1	1	1	1	1	−1	4	Moderate
Wang et al. (2010) (19)	1	1	−1	1	−1	1	1	1	1	−1	4	Moderate
Roy et al. (2010) (27)	1	1	−1	1	0	1	1	1	1	−1	5	Moderate
Heffner et al. (2011) (44)	1	1	−1	1	−1	1	1	1	1	−1	4	Moderate
Luchting et al. (2014) (45)	1	1	−1	1	−1	1	1	1	1	−1	4	Moderate
Queiroz et al. (2015) (46)	1	1	1	1	1	1	1	1	1	−1	8	Low
Luchting et al. (2016) (47)	1	1	−1	1	−1	1	1	1	1	−1	4	Moderate
Li et al. (2016) (48)	1	0	−1	−1	−1	1	1	1	1	−1	1	High
Degenhardt et al. (2017) (49)	1	1	−1	1	−1	1	1	1	1	−1	4	Moderate
Klyne et al. (2018) (40)	1	1	1	1	−1	1	1	1	1	1	8	Low
Tarabeih et al. (2019) (50)	1	1	1	1	−1	1	1	1	1	−1	6	Moderate
**HPA axis markers**												
Muhtz et al. (2013) (24)	1	1	−1	1	−1	1	1	1	1	−1	4	Moderate
Sveinsdottir et al. (2015) (26)	1	1	1	1	−1	1	1	1	1	1	8	Low

Q1: Was the research question or objective in this paper clearly stated? Q2: Was the study population clearly specified and defined? Q3: Was the participation rate of eligible persons at least 50%? Q4: Were all the subjects selected or recruited from the same or similar populations (including the same time period)? Were inclusion and exclusion criteria for being in the study prespecified and applied uniformly to all participants? Q5: Were a sample size justification, power description, or variance and effect estimates provided? Q6: For the analyses in this paper, were the exposure(s) of interest measured prior to the outcome(s) being measured? Q7: For exposures that can vary in amount or level, did the study examine different levels of the exposure as related to the outcome (e.g., categories of exposure or exposure measured as continuous variable)? Q8: Were the outcome measures (dependent variables) clearly defined, valid, reliable, and implemented consistently across all study participants? Q9: Were key potential confounding variables measured and adjusted statistically for their impact on the outcome(s)? Q10: Is the proportion of participants with missing data in the variable irrelevant (or is adequately justified to be irrelevant) or is it justified that statistical techniques to deal with missing data are appropriate (e.g., weighting adjustments or imputation methods)? NIHLBI, National Heart, Lung, and Blood Institute. Options: 1 = yes; 0 = unclear or cannot be determined; −1 = not reported. Total score range was 0–10: low risk of bias (8–10), medium (4–7), and high (≤3).

### Relationship between biomarker levels and NSLBP

We found 12 studies assessing immune-inflammatory biomarkers (n = 24) and two HPA biomarkers as assessed with cortisol (n = 2). **Table 3** summarizes findings for all the biomarkers explored in this systematic review.

**Table 3:** Synthesis of all identified evidence.

Biomarker	Immune Phenotype	Studies (n)	NSLBP (n)	HC(n)	Directionality NSLBP vs. HC (n, %)	Significant* differences NSLBP vs. HC (n, %)
IL-6	M1	7	321	228	↑ Higher levels (7/7, 100%)	(1/7, 14%)
IL-10	T_reg_/T_H_2	2	72	60	↑ Higher levels (1/2, 50%) ↓ Lower levels (1/2, 50%)	(1/2, 50%)
IL-17	T_H_17	1	37	25	↑ Higher levels (1/1, 100%)	(0/1, 0%)
IL-23	T_H_17	1	37	25	↑ Higher levels (1/1, 100%)	(1/1, 100%)
IL-1β	M1	3	161	81	↑ Higher levels (1/3, 33%)	(0/3, 0%)
					= Equal levels (1/3, 33%)	
					↓ Lower levels (1/3, 33%)	
TNF-α	M1	4	320	211	↑ Higher levels (4/4, 100%)	(1/4, 25%)
sTNF-R1	M1 (CIRS at high levels)	1	71	71	↑ Higher levels (1/1, 100%)	(1/1, 100%)
TGF-β	T_reg_	1	37	25	↑ Higher levels (1/1, 100%)	(1/1, 100%)
IFN-γ	T_H_1	1	37	25	↑ Higher levels (1/1, 100%)	(0/1, 0%)
GDF-15	T_reg_	1	556	522	↑ Higher levels (1/1, 100%)	(1/1, 100%)
CRP	M1/APP	2	142	62	↑ Higher levels (2/2, 100%)	(1/2, 50%)
hsCRP	M1/APP	2	52	1582	↑ Higher levels (1/2, 50%) ↓ Lower levels (1/2, 50%)	(0/2, 0%)
Cortisol	–	2	20	30	↑ Higher levels (1/2, 50%)	(2/2, 100%)
					↓ Lower levels (1/2, 50%)	

*p ≤ 0.5. Inflammatory macrophage M1, T helper (T_H_)1, and T_H_17 cytokines, coupled with acute phase proteins (APP) reactants including C-reactive protein (CRP) and high-sensitivity (hs)CRP, are pro-inflammatory and may be conceptualized as the immune-inflammatory response system (IRS). T regulatory (T_reg_) and T_H_2 cytokines are anti-inflammatory and part of the compensatory immune-regulatory system (CIRS). Further explanation of the function of the immune phenotypes can be found in the works of Andrés-Rodríguez et al. (32) and Maes and Carvalho (13).

#### Immune-inflammatory biomarkers

We found that growth differentiation factor 15 (GDF-15), IL-23, and soluble tumor necrosis factor receptor 1 (sTNF-R1) levels were significantly higher in individuals with NSLBP compared to HC. One study out of one (100%) for GDF-15 (50), IL-23 (45), and sTNF-R1 (46) showed significantly higher levels in NSLBP than HC, with strong (g = 0.87), moderate (g = 0.70), and low (g = 0.26) effect sizes, respectively. The RoB from these three studies was low or medium and the MQ was rated as medium (45, 50) or high (46). Results regarding GDF-15 and sTNF-R1 were related to NSLBP without specifying pain duration, whereas TGF-β and IL-23 results were regarding chronic NSLBP.

Two studies examined the association between IL-10 levels and chronic NSLBP (45, 48). One study (50%) reported significantly lower IL-10 levels in chronic NSLBP (48) in a sample of 70 participants (chronic NSLBP = 35, HC = 35), whereas the other study indicated a trend toward higher IL-10 levels in chronic NSLPB as compared with HC in a sample of 62 participants (chronic NSLBP = 37, HC = 25) (45). The study reporting significant differences presented a high RoB and a low MQ (48), whereas the study reporting non-significant differences showed a moderate RoB and a medium MQ (45). Although we found a trend of IL-6 high levels and TNF-α in NSLBP compared to HC, only one of the seven studies (14%) reported significant differences in IL-6 (48) in a sample of 70 participants (chronic NSLBP = 35, HC = 35) and one of the four studies (25%) reported significant differences in TNF-α (19) in a sample of 87 participants (chronic NSLBP = 58, HC = 29), both referring to chronic NSLBP. The RoB from these two studies was high (48) and moderate (19), and the MQ was rated as low (48) and high (19), respectively. Although results of TNF-α and IL-6 cytokines have only one study in each case with significant results, all studies assessing these biomarkers show a pattern of increased levels in NSLBP compared to HC.

In addition, two studies out of two found higher levels of CRP in NSLBP than in HC with no statistical consensus. Thus, only one of them (50%) that assessed participants with acute NSLBP found significant differences in a sample of 164 participants (acute NSLBP = 109, HC = 55) with a low effect size (g = 0.33) (40), whereas the non-significant results were captured in NSLBP without specifying the pain duration in a sample of 40 participants (NSLBP = 33, HC = 7) (49). In the first case, the RoB was low and the MQ was high (40), whereas the RoB was moderate and the MQ was medium in the study with non-significant differences (49).

We found no statistically significant differences for serum hsCRP in chronic NSLBP (27, 42), with one out of two studies not controlling for any confounder variables (27). Furthermore, we also found non-significant differences for IL-1β [chronic NSLBP (47, 49); acute NSLBP (40)], IL-17, and interferon gamma (IFN-γ) (chronic NSLBP) (45) in comparison to HC with only one study specifying pain duration (46). The RoB in these aforementioned studies was rated as low (40) or moderate (27, 42, 45, 47, 49) and the MQ was rated as high (40), medium (42, 45, 47, 49), or low (27).

#### HPA axis markers

Concerning HPA axis function, two studies assessed cortisol levels using salivary samples (24, 26). Although both studies exhibit significant results, one study found higher levels (26) and the other one found lower levels (24) of cortisol in NSLBP compared to HC. Both studies focused their results on chronic NSLBP, but their sample sizes varied from 1,150 (chronic NSLBP = 305, HC = 845) (24) to 53 participants (chronic NSLBP = 20, HC = 33) (26), respectively. The RoB was low (26) and moderate (24) and the MQ was low (24) and medium (26). To sum up, we found evidence of altered cortisol levels in NSLBP compared to HC.

**Figure 2:**
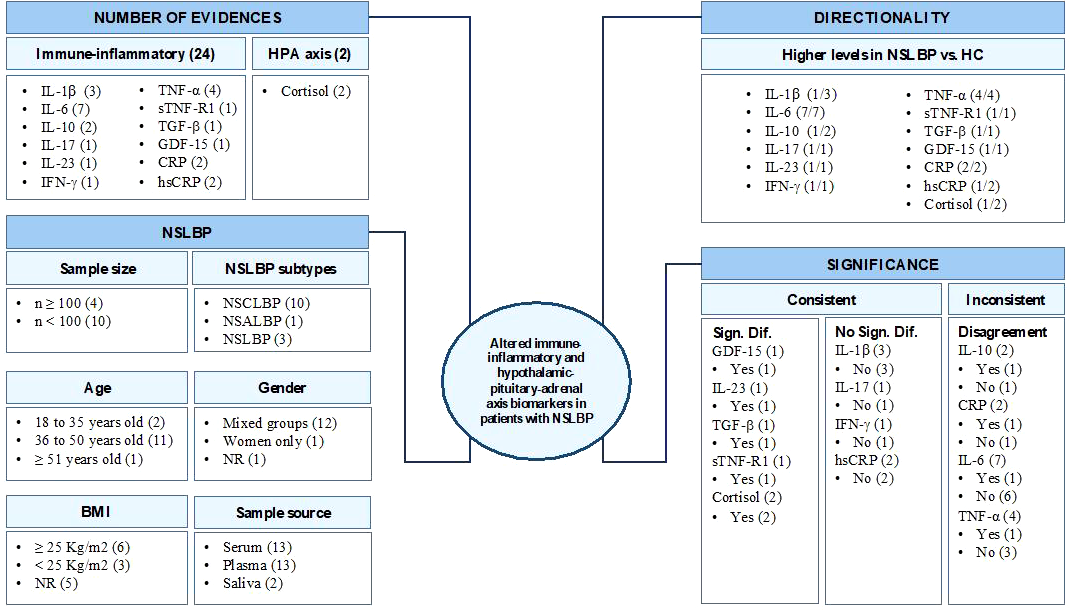
IL, interleukin; IFN, interferon; TNF, tumor necrosis factor; TGF, transforming growth factor; GDF, growth differentiation factor; CRP, C-reactive protein; hsCRP, high-sensitivity CRP; NSCLBP, non-specific chronic low back pain; NSALBP, non-specific acute low back pain; NSLBP, non-specific low back pain; NR, not reported; BMI, body mass index (kg/m^2^); HC, healthy controls.

**Table 4** and **Figure 2** summarize the findings on immune-inflammatory and HPA axis biomarkers in NSLBP.

**Table 4:** Summary of studies’ results on immune-inflammatory and HPA axis biomarkers (*n* = 14).

Author (year)	Diagnosis	Mean pain	*n*		Biomarker, Mean (SD)			
			NSLBP	HC	NSLBP	HC	*p*	*g*
**IL-6 (n = 7)**								
Roy et al. (2010) (27)	NSCLBP	NR	11	10	3.97 pg/ml (0.44)	3.12 pg/ml (0)	.06	0.87
Heffner et al. (2011) (44)	NSCLBP	NR	25	25	1.2 pg/ml (1.0)	1.1 pg/ml (0.6)	.67	0.12
Luchting et al. (2014) (45)	NSCLBP	3.37^a^	37	25	2.1 pg/ml (NR)	1.4 pg/ml (NR)	>.05	NR
Queiroz et al. (2015) (46)	NSLBP	5.31	71	71	2.25 pg/ml (1.80)	1.63 pg/ml (3.67)	.37	0.21
Li et al. (2016) (48)	NSCLBP	NR	35	35	170% level	100% level	**<.05**	NA
Degenhardt et al. (2017) (49)	NSLBP	4^a^	33	7	0 pg/ml (11.30)	0 pg/ml (0.07)	09	0
Klyne et al. (2018) (40)	NSALBP	NR	109	55	0.8 pg/ml (1.58)	0.7 pg/ml (0.54)	.24	0.07
**IL-10 (n = 2)**								
Luchting et al. (2014) (45)	NSCLBP	3.37^a^	37	25	4.1 pg/ml (NR)	3.6 pg/ml (NR)	>.05	NR
Li et al. (2016) (48)	NSCLBP	NR	35	35	75% level	100% level	**<.01**	NR
**IL-17 (n = 1)**								
Luchting et al. (2014) (45)	NSCLBP	3.37^a^	37	25	4.3 pg/ml (NR)	4.0 pg/ml (NR)	>.05	NR
**IL-23 (n = 1)**								
Luchting et al. (2014) (45)	NSCLBP	3.37^a^	37	25	1.21 pg/ml (0.43)	0.94 pg/ml (0.29)	**<.01**	0.70
**IL-1β (n = 3)**								
Luchting et al. (2016) (47)	NSCLBP	3.5	19	19	1. 9 NR/NR (NR)	1.8 NR/NR (NR)	>.05	NR
Degenhardt et al. (2017) (49)	NSLBP	4^a^	33	7	0 pg/ml (0.03)	0 pg/ml (0.03)	.28	0
Klyne et al. (2018) (40)	NSALBP	NR	109	55	0.2 pg/ml (0.24)	0.3 pg/ml (0.31)	.20	0.37
**TNF-α (n = 4)**								
Wang et al. (2008) (43)	NSCLBP	5.8	120	120	57.6% positive	12.3% positive	>.05	NR
Wang et al. (2010) (19)	NSCLBP	5.19b	58	29	2.58 pg/ml (NR)	0.1 pg/ml (NR)	**<.01**	NR
Degenhardt et al. (2017) (49)	NSLBP	4^a^	33	7	3.68 pg/ml (4.28)	0.60 pg/ml (2.92)	.23	0.74
Klyne et al. (2018) (40)	NSALBP	NR	109	55	1.1 pg/ml (1.53)	1.0 pg/ml (0.76)	.24	0.07
**sTNF-R1 (n = 1)**								
Queiroz et al. (2015) (46)	NSLBP	5.31	71	71	1275 pg/ml (404.18)	1087 pg/ml (448.87)	**.02**	0.26
**TGF-b (n = 1)**								
Luchting et al. (2014) (45)	NSCLBP	3.37a	37	25	0.21 (0.07)	0.14 (0.05)	**.01**	1.10
**IFN-γ (n = 1)**								
Luchting et al. (2014) (45)	NSCLBP	3.37a	37	25	4.19 (3.54)	3.60 (2.20)	>.05	0.20
**GDF-15 (n = 1)**								
Tarebeith et al. (2019) (50)	NSLBP	NR	556	522	2.65 NR/NR (0.08)	2.58 NR/NR (0.08)	**<.01**	0.87
**CRP (n = 2)**								
Degenhardt et al. (2017) (49)	NSLBP	4^a^	33	7	0.82 μg/ml (3.45)	0.65 μg/ml (6.96)	.71	0.04
Klyne et al. (2018) (40)	NSALBP	NR	109	55	1.8 μg/ml (2.60)	1.0 μg/ml (1.83)	**<.01**	0.33
**hsCRP (n = 2)**								
Gebhardt et al. (2006) (42)	NSCLBP	4.9^a^	41	1572	1.3 ug/ml (1.39)	1.49 ug/ml (NR)	>.05	NR
Roy et al. (2010) (27)	NSCLBP	NR	11	10	2.50 ug/ml (0.79)	1.05 ug/ml (0.34)	.11	0.75
**Cortisol (n = 2)**								
Muhtz et al. (2013) (24)	NSCLBP	6.03^a^	20	33	2.25 mg/l (NR)	3.35 mg/l (NR)	**<.01**	NR
Sveinsdottir et al. (2015) (26)	NSCLBP	NR	305	845	7.63 nmol/l (7,26)	4.23 nmol/l (24,26)	**.02**	NR

HC, healthy controls; NSCLBP, non-specific chronic low back pain; NSALBP, non-specific acute low back pain; NR, not reported; NA, not applicable; CRP, C-reactive protein; hsCRP, high-sensitivity CRP; IL, interleukin; TNF, tumor necrosis factor; GDF, growth differentiation factor. a, Visual Analogue Scale (VAS), score 0-10. Bold, statistically significant differences compared to HC.

Bold, statistically significant differences compared to HC.

## Discussion

To the best of our knowledge, this is a pioneering systematic review focusing on alterations in immune-inflammatory and HPA axis biomarker levels in individuals with NSLBP compared to HC. Although previous reports showed that alterations in levels of IL-6 (28, 30), TNF-α (28–30), and CRP (28, 30) were associated with NSLBP and acute NSLBP (29), the results of our systematic review are not completely in line with previous findings when using exclusively HC as a comparison group. For the first time, preliminary and limited evidence of increased levels of sTNF-R1 (46), GDF-15 (50), and IL-23 (45) was found in NSLBP compared to HC, although this evidence was derived from only one study for each biomarker.

The soluble receptor sTNF-R1 binds pro-inflammatory cytokine TNF-α. This biomarker plays the main role in regulating TNF-α *in vivo* activity and its circulating levels are increased in inflammatory diseases. At low or intermediate concentrations, the sTNF-R1 preserves and enhances the TNF-α activity, whereas, at high levels, it inhibits the TNF-α activity protecting from an excessive inflammatory response (51). It has been described that TNF-α causes muscular catabolism (52) and loss of bone by osteoclasts activation and inhibition that could be related to chronic pain. The high levels of sTNF-R1 found in chronic NSLBP participants (46) might play a compensatory response role to previous high TNF-α levels (13).

The peptide hormone GDF-15 belongs to the TGF (TGF-β) superfamily and is expressed in multiple tissues mainly after tissue damage, apoptosis, inflammation, and oxidative stress. Under normal physiological conditions, TGF-β expression is low, with a role in recovering and reorganizing muscle structure (53), whereas, under many inflammatory pathological conditions, it is overexpressed due to its protective role. It remains uncertain whether GDF-15 increased levels observed in chronic NSLBP is a causative mediator or a risk biomarker.

The pro-inflammatory cytokine IL-23 is mainly produced by dendritic cells and macrophages. IL-23 favors the synthesis of pro-inflammatory cytokines IL-17 and IL-22 by promoting the expansion of T_H_17 cells (54). High circulating levels of IL-23 have been found in chronic NSLBP compared to HC, but with a diminished expression in T cells (45). In individuals with chronic pain, a decrease in T_H_17/T_reg_ cells ratio and IL-23 expression, as well as an increase in TGF-β in T cells, has been found. It is significant to note that, in these individuals, the TNF-α, IFN-γ, and IL-4 circulating levels were undetectable (45). These results point to an association between chronic NSLBP and immune suppression more than an activation as it has been observed in other chronic diseases (55). The question is whether this disturbed balance is (1) a consequence of the long-time pain inducing the dysregulation of the immune system to an anti-inflammatory phenotype or (2) the dysregulation existing previously and predisposing to suffer chronification of pain symptoms because treated individuals who improved in their pain condition continue to show the same imbalance (45).

The results for sTNF-R1, GDF-15, TGF-β, and IL-23 should be interpreted with caution because only one study was available for each biomarker. Nonetheless, findings from GDF-15 comes from one study with 1,078 participants (50), whereas sTNF-R1, TGF-β, and IL-23 results have sample sizes ranging from 62 [Luchting et al. (45) for TGF-β and IL-23] to 142 participants [Queiroz et al. (46) for sTNF-R1]. The study that reported higher sTNF-R1 levels in NSLBP (46) included only elderly women (from 65 to 82 years old), and therefore, these results could be related to the aging process or to gender issues. Furthermore, the IL-23 and TGF-β findings appeared to be driven exclusively by chronic NSLBP (45), whereas findings related to GDF-15 and sTNF-R1 were pointing out NSLBP. The RoB of the three latter studies was moderate (45, 50) or low (46) and the MQ was medium (45, 50) and high (46).

CRP alterations (28, 30) in NSLBP and in acute NSLBP (29) were partially replicated in the present work. Although two studies found an absolute elevation of CRP levels in NSLBP compared to HC, only one study reported statistically significant differences with a low effect size in acute NSLBP (40). This latter study yielded low RoB and high MQ (40) with a total of 70 participants, whereas the non-significant study (49) showed moderate RoB and medium MQ. The small sample size of the last study (n = 40) and its target population (chronic NSLBP) may explain these results discrepancies through unpowerful statistical results without significant conclusions. Previous systematic research clarifies how high levels of pain intensity are correlated with CRP increased levels (28, 30) in NSLBP and acute NSLBP (29), whereas the present study emphasized how the target population (i.e., acute NSLBP) is the key detonator to exhibit significant results in a worsening dysregulation of CRP level expression. In congruence with significant results in NSLBP (30), we did not find evidence for alterations in hsCRP, with two out of two studies showing non-significant results for serum hsCRP (27, 42). Studies reporting hsCRP results were congruent with the condition (i.e., chronic NSLBP) and they were composed of sample sizes of 1,613 and 21 participants (27, 42) with moderate RoB and medium (42) to low (27) MQ. Potential adaptive mechanisms underlying CRP/hsCRP differences can be explained by acute NSLBP and chronic NSLBP targets.

In the same way, the current systematic review found inconsistent evidence for IL-10, IL-6, and TNF-α alterations in NSLBP. Specifically, IL-10 was significantly lower in one study (48) and non-significantly increased in another study (45) compared to HC. IL-10 is essential for the homeostasis of the immune system by playing an anti-inflammatory role effect inhibiting pro-inflammatory cytokines such as TNF-α, IL-6, and IL-1 (56, 57). However, as literature shows, low levels of IL-10 are presented in chronic widespread pain (57). This fact may point out a pro-inflammatory state that could promote tissue inflammation. The scientific evidence indicated that IL-10 data should be interpreted with caution because studies assessing this biomarker showed high (48) to moderate (45) RoB and low (48) to medium (45) MQ, although both studies were aiming for chronic NSLBP. Moreover, these studies recruited small samples, from 62 (45) to 70 (48) participants, which limits the interpretation of their findings and makes it difficult to make solid conclusions.

IL-6 and TNF-α pro-inflammatory cytokines are important neuromodulators in central sensitization by the activation of glial cells (astrocytes and microglia) within the spinal cord inducing hyperalgesia and allodynia (58). Even though higher levels of IL-6 and TNF-α appeared to increase in NSLBP compared to HC, solid conclusions cannot be established. Only one out of seven studies reported statistically significant differences in IL-6 (48) and only one out of four reports observed statistically significant differences in TNF-α (19) regarding chronic NSLBP in both findings. The RoB from these two studies was high (48) and moderate (19) and the MQ low (48) and high (19), respectively. In contrast to previous reviews (28–30), we conclude that there is inconclusive evidence for significantly higher levels of IL-6 and TNF-α in NSLBP compared to HC. The low presence of significant results in our review compared to previous reviews may be explained by our use of HC as a comparator group as well as by target condition heterogeneity. For example, four IL-6 studies were conducted including chronic NSLBP in which one reported significant evidence, one included NSLBP participants, and another used acute NSLBP. In TNF-α biomarker studies, two studies focused on chronic NSLBP with one reporting significant results, whereas one focused on NSLBP and another on acute NSLBP.

In agreement with previous reviews, we found no evidence for alterations in IL-1β, IL-17, and IFN-γ. Of these, IL-1β showed most clearly non-significant results, with three out of three studies reporting non-significance for serum IL-1β (40, 47, 49). The three studies assessing IL-1β targeted chronic NSLBP without specifying pain duration (47, 49) and acute NSLBP (40).

Regarding HPA functioning, the two identified studies found significantly altered levels of salivary cortisol in chronic NSLBP compared to HC, with low (26) or moderate RoB (24) and medium (26) or low MQ (24). The sample sizes of these studies varied from 53 to 1,150 participants. As there are age-dependent changes in HPA axis function, both studies controlled age as a potentially confounding variable in the analyses. However, both studies found significant differences: the larger study (26) supports the fact that cortisol levels are increased in NSLBP, whereas Muhtz et al. (24) suggest decreased levels in chronic NSLBP. Agreeing with Juruena et al. (22), being exposed to a pain condition (constant or not) is a potential stressor that activates HPA functioning through cortisol levels, but being exposed to a chronic pain condition yields to dysfunction in cortisol response that can endanger the HPA system. Further research is needed to target the direction of these neuroendocrine mechanisms and their relationship with NSLBP.

The identification of signature immune-inflammatory and HPA axis biomarkers in NSLBP could provide new targets for pharmacological therapy. Furthermore, to the extent that variations in the levels of these biomarkers could be related to the response of patients to the different treatments (pharmacological and/or psychological) available, they could provide clinicians with objective measures to guide therapeutic choices along the lines of personalized medicine for this highly treatment-resistant condition.

### Limitations and strengths

Our findings must be interpreted taking the following limitations and strengths into account. First, the small amount of evidence found for most explored biomarkers as well as the limited sample sizes of some studies made it difficult to report strong conclusions about the immune-inflammatory pathways underpinning NSLBP. Second, the moderate RoB (about 71%) and the medium (50%) to low MQ (about 21%) of the studies included in this systematic review could lead to imprecise conclusions. Third, results of our heterogeneity analysis indicated that a considerable number of studies (about 45%) did not control at least 10 of the 20 established confounding variables. All of these 20 potential confounder variables are depicted in the ICS (**Appendix B**). Fourth, sample source variety can yield to provide misleading results: nine studies used serum, two used plasma, one used serum and plasma, and two studies used salivary cortisol. Fifth, given the characteristics of the studies, it was impossible to perform a complementary analysis between biomarkers and the NSLBP type (acute, subacute, and chronic), as well as taking into account clinical variables that have been associated with pain in previous studies (e.g., pain intensity, depression, sleep quality, smoking, and medications). Medication, in particular, is a confounding variable that is difficult to disentangle from the pain disorder itself in this type of research. Possible intake not only of pain medication, in particular medications that have a clear impact on the biomarkers analyzed, including analgesics, NSAIDs, corticosteroids, and opioids, but also of antidepressants and mood stabilizers needs to be taken into account, given the high comorbidity of pain disorders with mood disorders such as depression and anxiety (59). The strategies usually employed to address this problem involve a medication washout or statistical control. In our review, we have assessed these aspects of the MQ of the included studies using the ICS scale (Appendices B and C). Finally, only published studies in English or Spanish were included in this study, so other evidence could have been omitted. Nevertheless, we included additional search sources such as the review of references from other studies, review of references from other systematic reviews, and gray literature.

The strengths of this study are the number of databases explored, compliance with PRISMA guidelines, prospective registration in PROSPERO, preliminary review and validation of the Boolean searches according to PRESS guidelines, the inclusion of Rayyan as a tool to minimize possible loss of evidence, and consensual review between two reviewers in the different phases of screening and extraction of the data. In addition, the inclusion of RoB and MQ tools, consideration of an exclusive healthy comparison group, and selection of baseline data not previously reported in other systematic reviews contribute to generate an additional value to the limited scope of our findings. Longitudinal studies are required to establish cause and effects to evaluate the predictive value of these biomarkers.

## Conclusions

In contrast to previous systematic reviews, our results indicate that there is not enough evidence to conclude that CRP, IL-6, and TNF-α levels are altered in NSLBP in comparison to HC. This study showed preliminary and limited evidence (only one study for each biomarker) that GDF-15, IL-23, and sTNF-R1 are significantly increased in NSLBP as well as TGF-β in chronic NSLBP compared to HC, altogether suggesting a pro-inflammatory state in NSLBP. Regarding the anti-inflammatory cytokine IL-10, lower levels, although with inconsistent evidence, were found in chronic NSLBP. Preliminary evidence for IL-17 and IFN-γ in chronic NSLBP was not found. IL-1β and hsCRP showed non-significant associations with NSLBP. Pro-inflammatory biomarkers are altered due to the pain exposure, whereas an anti-inflammatory response is presented as a consequence of the inflammation process in chronic pain situations. Regarding cortisol, one study showed an increase and the other a decrease in salivary levels in NSLBP compared to HC. However, with the incongruent evidence regarding the directionality of cortisol response, both studies indicate a dysregulation of HPA functioning due to constant pain exposure. To sum up, 10 out of 14 studies reported their findings on chronic NSLBP, so the current results could be partially relaying under chronic condition. Overall, the number of available studies, RoB, and MQ limited the interpretation of these results. Consequently, more scientific evidence is needed to confirm and quantify the magnitude of the findings reported in the current systematic review, as well as to better understand the role of these specific biomarkers under NSLBP condition.

## Data availability statement

The original contributions presented in the study are included in the article/**Supplementary Material**. Further inquiries can be directed to the corresponding authors.

## Author contributions

JL, AS, SE, AF-S, and XB designed the study. JS-M, and MC-C performed the eligibility criteria, data extraction, and study coding. JS-M, and AC-C performed the data analysis, synthesized all extracted data, and drafted the manuscript. MG and ME realized a value contribution specifically in the introduction, discussion, and conclusion section regarding biological point of view as well as revised and approved the final version of the manuscript. MM critically revised and supervised the final draft. All authors commented on, revised, and approved the draft and the final manuscript.

## Funding

This work was supported by the Institute of Health Carlos III (ISCIII; PI19/00112) cofinanced with European Union ERDF funds. JS-M has a PFIS predoctoral contract from the ISCIII (FI20/00034). AC-C has a FI predoctoral contract from AGAUR (FI_B/00216). AF-S acknowledges the funding from the Serra Húnter program (Generalitat de Catalunya; reference number UAB-LE-8015). The funding sources and sponsors had no influence on the design of the study, data collection and analysis, or the writing of the manuscript.

## Acknowledgments

We thank Dr. Alessandro Chiarotto for his valuable comments on a previous draft of this manuscript.

## Conflict of interest

The authors declare that the research was conducted in the absence of any commercial or financial relationships that could be construed as a potential conflict of interest.

## Publisher’s note

All claims expressed in this article are solely those of the authors and do not necessarily represent those of their affiliated organizations, or those of the publisher, the editors and the reviewers. Any product that may be evaluated in this article, or claim that may be made by its manufacturer, is not guaranteed or endorsed by the publisher.

## Abbreviations

HC, Healthy control; ICS, Immune confounders scale; IRS, Immune-inflammatory response system; MQ, Methodological quality; NSLBP, Non-specific low back pain; RoB, Risk of bias.
