# Supplementary material for: Immune-inflammatory and hypothalamic-pituitary-adrenal axis biomarkers are altered in patients with non-specific low back pain: A systematic review
Source: Front Immunol. 2022 Sep 2;13:945513. doi: 10.3389/fimmu.2022.945513 (PMC9478440; doi:10.3389/fimmu.2022.945513)
Supplement: Supplementary file 1 [file DataSheet_1.docx]

***Supplementary Material***

**Appendix A**

**TABLE S1|** Specific search for each database.

| **PubMed (*n* = 838)** |
| --- |
| ("back pain"[MeSH Terms] OR "low back pain"[MeSH Terms] OR “lumbar vertebrae”[MeSH Terms] OR “sciatica”[MeSH Terms] OR "radiculopathy"[MeSH Terms] OR "back pain"[Title] OR “lumbar vertebrae”[Title] OR “sciatica”[Title] OR “radiculopathy”[Title]) AND ("inflammation"[MeSH Terms] OR "inflammation mediators"[MeSH Terms] OR inflamm*[Title] OR "cytokines"[MeSH Terms] OR "cytokines"[All Fields] OR "chemokine"[All Fields] OR "interleukin"[All Fields] OR "interferon"[All Fields] OR "tumor necrosis factors"[MeSH Terms] OR "tumor necrosis factor"[All Fields] OR "transforming growth factor beta"[MeSH Terms] OR "transforming growth factor"[All Fields] OR "c-reactive protein"[MeSH Terms] OR "c-reactive protein"[All Fields] OR "lymphocytes"[MeSH Terms] OR "lymphocytes"[All Fields] OR "macrophages"[MeSH Terms] OR "macrophages"[All Fields] OR "microglia"[MeSH Terms] OR "microglia"[All Fields] OR "leukocytes"[MeSH Terms] OR leukocyte*[All Fields] OR "neutrophil"[All Fields] OR "eosinophil"[All Fields] OR "basophil"[All Fields] OR "monocyte"[All Fields] OR "mast cells"[MeSH Terms] OR "mast cells"[All fields] OR "dendritic cells"[MeSH Terms] OR "dendritic"[All fields] OR "hydrocortisone"[MeSH Terms] OR "cortisol"[All Fields] OR "hypothalamo-hypophyseal system"[MeSH Terms] OR "hypothalamic–pituitary–adrenal"[All Fields] OR "adrenocorticotropic hormone"[MeSH Terms] OR "adrenocorticotropic hormone"[All Fields] OR "corticotropin-releasing hormone"[MeSH Terms] OR "corticotropin-releasing hormone"[All Fields] OR "dexamethasone"[MeSH Terms] OR "dexamethasone"[All Fields] OR "glucocorticoids"[MeSH Terms] OR "glucocorticoids"[All Fields] OR "miner-alocorticoid"[All Fields]) |
| **Medline(R) (*n* = 660)** |
| (MH=(back pain) OR MH=(low back pain) OR MH=(lumbar vertebrae) OR MH=(sciatica) OR MH=(radiculopathy) OR TI=(back pain) OR TI=(lumbar vertebrae) OR TI=(sciatica) OR TI=(radiculopathy)) AND (MH=(inflammation) OR MH=(inflammation mediators) OR TI=(inflamm*) OR MH=(cytokines) OR TS=(cytokines) OR TS=(chemokine) OR TS=(interleukin) OR TS=(interferon) OR MH=(tumor necrosis factors) OR TS=(tumor necrosis factor) OR MH=(transforming growth factor beta) OR TS=(transforming growth factor) OR MH=(c-reactive protein) OR TS=(c-reactive protein) OR MH=(lymphocytes) OR TS=(lymphocytes) OR MH=(macrophages) OR TS=(macrophages) OR MH=(microglia) OR TS=(microglia) OR MH=(leukocytes) OR TS=(leukocyte*) OR TS=(neutrophil) OR TS=(eosinophil) OR TS=(basophil) OR TS=(monocyte) OR MH=(mast cells) OR TS=(mast cells) OR MH=(dendritic cells) OR TS=(dendritic) OR MH=(hydrocortisone) OR TS=(cortisol) OR MH=(hypothalamo-hypophyseal system) OR TS=(hypothalamic–pituitary–adrenal) OR MH=(adrenocorticotropic hormone) OR TS=(adrenocorticotropic hormone) OR MH=(corticotropin-releasing hormone) OR TS=(corticotropin-releasing hormone) OR MH=(dexamethasone) OR TS=(dexamethasone) OR MH=(glucocorticoids) OR TS=(glucocorticoids) OR TS=(miner-alocorticoid)) |
| **Web of Science (*n* = 347)** |
| (TI=(back pain) OR TI=(lumbar vertebrae) OR TI=(sciatica) OR TI=(radiculopathy)) AND (TI=(inflamm*) OR TS=(cytokines) OR TS=(chemokine) OR TS=(interleukin) OR TS=(interferon) OR TS=(tumor necrosis factor) OR TS=(transforming growth factor) OR TS=(c-reactive protein) OR TS=(lymphocytes) OR TS=(macrophages) OR TS=(microglia) OR TS=(leukocyte*) OR TS=(neutrophil) OR TS=(eosinophil) OR TS=(basophil) OR TS=(monocyte) OR TS=(mast cells) OR TS=(dendritic) OR TS=(cortisol) OR TS=(hypothalamic–pituitary–adrenal) OR TS=(adrenocorticotropic hormone) OR TS=(corticotropin-releasing hormone) OR TS=(dexamethasone) OR TS=(glucocorticoids) OR TS=(miner-alocorticoid)) |
| **PsycINFO (*n* = 103)** |
| (MA back pain OR MA low back pain OR MA lumbar vertebrae OR MA sciatica OR MA radiculopathy OR TI back pain OR TI lumbar vertebrae OR TI sciatica OR TI radiculopathy) AND (MA inflammation OR MA inflammation mediators OR TI inflamm* OR MA cytokines OR TX cytokines OR TX chemokine OR TX interleukin OR TX interferon OR MA tumor necrosis factors OR TX tumor necrosis factor OR MA transforming growth factor beta OR TX transforming growth factor OR MA c-reactive protein OR TX c-reactive protein OR MA lymphocytes OR TX lymphocytes OR MA macrophages OR TX macrophages OR MA microglia OR TX microglia OR MA leukocytes OR TX leukocyte* OR TX neutrophil OR TX eosinophil OR TX basophil OR TX monocyte OR MA mast cells OR TX mast cells OR MA dendritic cells OR TX dendritic OR MA hydrocortisone OR TX cortisol OR MA hypothalamo-hypophyseal system OR TX hypothalamic–pituitary–adrenal OR MA adrenocorticotropic hormone OR TX adrenocorticotropic hormone OR MA corticotropin-releasing hormone OR TX corticotropin-releasing hormone OR MA dexamethasone OR TX dexamethasone OR MA glucocorticoids OR TX glucocorticoids OR TX miner-alocorticoid) |
| **Scopus (*n* = 35)** |
| (ALL (back pain OR lumbar vertebrae OR sciatica OR radiculopathy) AND ALL (inflamm* OR cytokines OR chemokine OR interleukin OR interferon OR tumor necrosis factor OR transforming growth factor OR c-reactive protein OR lymphocytes OR macrophages OR microglia OR leukocyte* OR neutrophil OR eosinophil OR basophil OR monocyte OR mast cells OR dendritic OR cortisol OR hypothalamic–pituitary–adrenal OR adrenocorticotropic hormone OR corticotropin-releasing hormone OR dexamethasone OR glucocorticoids OR miner-alocorticoid)) |

*Note.* The following filters were activated in all databases if possible: publication date (from 2005/01/01 to 2021/11/04), type of publication (only studies of interest), species (humans), and languages (English and Spanish).

**Appendix B**

**TABLE S2 |** Immune confounders scale (ICS).

|  | **Methodological quality of the study** |  |
| --- | --- | --- |
| 1 | Study sample ≥ 128 participants including patients and HC (1= Yes, 0 = No) |  |
| 2 | Did the study control results for potential confounders (e.g. age, BMI, gender, race)? (1= Yes, 0 = No) |  |
| 3 | Were participants with FM and HCs age- and-gender-matched or statistically controlled? (1= Yes, 0 = No) |  |
| 4 | Was the time of sample collection specified (e.g. morning vs. evening)? (1= Yes, 0 = No) |  |
| 5 | Were participants with FM free of immunomodulatory drugs including anti-cytokines, corticoids, immunoglobulins, and immunosuppressants, been through a medication washout or the intake was statistically controlled? (1= Yes, 0 = No) |  |
| 6 | Were participants with FM free of antidepressants and mood stabilizers or statistically controlled? (1= Yes, 0 = No) |  |
| 7 | Reporting of either the manufacturer of the test or its parameters (detection limit and coefficient of variation) (1= Yes, 0 = No) |  |
| 8 | Reporting how data under detection limit was handled (1 = Yes, 0 = No) |  |
| 9 | Reporting % of the sample under detection limit (1=Yes, 0= No) |  |
| 10 | Reporting blood fraction (serum, plasma, culture supernatant or whole blood) (1= Yes, 0 = No) |  |
|  | **Cytokine confounders red points**  *The red points should not be given if the item is statistically controlled* |  |
| 1 | 3 red points for comorbid illnesses such as autoimmune disorders & other immune disorders including RA, psoriasis, IBD, COPD, MS |  |
| 2 | 3 red points for use of recreational drugs such as methamphetamine or opioids (Not applicable if psychiatric disorders are excluded) |  |
| 3 | 2 red points for comorbidity with MDD / BD (Not applicable if psychiatric disorders are excluded) |  |
| 4 | 2 red points when groups were not matched for age |  |
| 5 | 2 red points for sex |  |
| 6 | 2 red points for medication use as for example immunomodulators |  |
| 7 | 2 red points for early traumatic life events |  |
| 8 | 2 red points for shift work and primary sleep disorders |  |
| 9 | 1.5 red points for antidepressants |  |
| 10 | 1 red point for other neuro-psychiatric comorbidities, as for example schizophrenia, autism, GAD, PTSD |  |
| 11 | 1 red point for more common systemic immune disorders including diabetes type 1/2, essential hypertension, metabolic syndrome |  |
| 12 | 1 red point for not fasting (8 hours before blood extraction) |  |
| 13 | 1 red point for use of omega-3 and antioxidant supplements |  |
| 14 | 1 red point for BMI |  |
| 15 | 1 red point for physical activity or sedentary life |  |
| 16 | 1 red point for smoking |  |
| 17 | 1 red point for use of oral contraceptives or NSAIDs |  |
| 18 | 0.5 red points for ethnicity in countries such as US, Brazil (not China or Japan) |  |
| 19 | 0.5 red points for seasonality |  |
| 20 | 0.5 red points for diurnal variation (8-10 a.m. versus all other time points) |  |

*Note:* Threshold of study samples is stablished as it is the minimum needed for statistical power. Cytokine confounders red points should be given when the item is not reported (or statistically controlled).

**Appendix C**

**TABLE S3 |** Methodological quality assessment of included studies (*n* = 14).

| **Study** | **Immune Confounders Scale (ICS)** | | | | | | | | | | **Score** | **MQ** |
| --- | --- | --- | --- | --- | --- | --- | --- | --- | --- | --- | --- | --- |
|  | **Q1** | **Q2** | **Q3** | **Q4** | **Q5** | **Q6** | **Q7** | **Q8** | **Q9** | **Q10** |  |  |
| **Immune-inflammatory biomarkers** | | | | | | | | | | | | |
| Gebhardt et al. (2006) [42] | 1 | 1 | 1 | 0 | 1 | 0 | 1 | 1 | 0 | 1 | 7 | Medium |
| Wang et al. (2008) [43] | 1 | 1 | 1 | 1 | 1 | 1 | 1 | 1 | 0 | 1 | 9 | High |
| Wang et al. (2010) [19] | 0 | 1 | 1 | 1 | 1 | 1 | 1 | 1 | 0 | 1 | 8 | High |
| Roy et al. (2010) [27] | 0 | 0 | 1 | 0 | 0 | 0 | 1 | 1 | 0 | 1 | 4 | Low |
| Heffner et al. (2011) [44] | 0 | 1 | 1 | 1 | 0 | 1 | 1 | 1 | 0 | 1 | 7 | Medium |
| Luchting et al. (2014) [45] | 0 | 1 | 1 | 0 | 1 | 0 | 1 | 1 | 0 | 1 | 6 | Medium |
| Queiroz et al. (2015) [46] | 1 | 1 | 1 | 1 | 1 | 1 | 1 | 1 | 0 | 1 | 9 | High |
| Luchting et al. (2016) [47] | 0 | 1 | 1 | 1 | 1 | 0 | 1 | 1 | 0 | 1 | 7 | Medium |
| Li et al. (2016) [48] | 0 | 0 | 0 | 0 | 0 | 0 | 1 | 1 | 0 | 1 | 3 | Low |
| Degenhardt et al. (2017) [49] | 0 | 0 | 1 | 0 | 1 | 0 | 1 | 1 | 0 | 1 | 5 | Medium |
| Klyne et al. (2018) [40] | 1 | 1 | 1 | 1 | 1 | 1 | 0 | 1 | 1 | 1 | 9 | High |
| Tarebeith et al. (2019) [50] | 1 | 1 | 1 | 0 | 0 | 0 | 1 | 1 | 0 | 1 | 6 | Medium |
| **HPA axis markers** | | | | | | | | | | | | |
| Muhtz et al. (2013) [24] | 0 | 0 | 0 | 1 | 0 | 0 | 1 | 1 | 0 | 1 | 4 | Low |
| Sveinsdottir et al. (2015) [26] | 1 | 0 | 0 | 1 | 1 | 1 | 1 | 1 | 0 | 1 | 7 | Medium |

*Note.* MQ: methodological quality. Q1: Study sample ≥ 128 participants including patients with NSLBP and HC? Q2: Did the study control results for potential confounders (e.g., age, BMI, gender, race)? Q3: Were participants with NSLBP and HC age- and-gender-matched or statistically controlled? Q4: Was the time of sample collection specified (e.g., morning vs. evening)? Q5: Were participants with NSLBP free of immunomodulatory drugs including anti-cytokines, corticoids, immunoglobulins, and immunosuppressants, been through a medication washout or the intake was statistically controlled? Q6: Were participants with NSLBP free of antidepressants and mood stabilizers or statistically controlled? Q7: Reporting of either the manufacturer of the test or its parameters (detection limit and coefficient of variation)? Q8: Reporting how data under detection limit was handled? Q9: Reporting % of the sample under detection limit? Q10: Reporting blood fraction (serum, plasma, culture supernatant or whole blood? Total score range was 0-10: low MQ (0-4), medium (5-7), and high (8-10). Options: 1 = yes; 0 = unclear, not reported, or cannot be determined.
